# Case Report: Synchronous ALK-positive laryngeal inflammatory myofibroblastic tumor and EGFR-mutant lung adenocarcinoma in a child

**DOI:** 10.3389/fped.2026.1874110

**Published:** 2026-08-06

**Authors:** Guodun Zheng, Dengke Luo, Taozhen He, Miao Yuan, Gang Yang, Shiyi Dai, Chang Xu

**Affiliations:** Department of Pediatric Surgery, West China Hospital of Sichuan University, Chengdu, Sichuan Province, China

**Keywords:** ALK rearrangement, case report, EGFR exon 20 insertion, inflammatory myofibroblastic tumor, lung adenocarcinoma, next-generation sequencing, pediatric oncology, synchronous primary tumors

## Abstract

The concurrent occurrence of two histogenetically distinct tumors in a child is exceptionally uncommon and may create substantial diagnostic uncertainty. To the best of our knowledge, this is the first reported case of synchronous diagnosis of an ALK-rearranged inflammatory myofibroblastic tumor (IMT) of the larynx and EGFR-mutant lung adenocarcinoma. A 7-year-old boy presented with dyspnea and hoarseness. A laryngeal mass was resected and confirmed to be ALK-positive IMT harboring a THBS1-ALK rearrangement. PET-CT also detected a solitary lung nodule in the right lower lobe, which was initially considered possible metastatic IMT. However, serial chest CT over 22 months demonstrated only minimal, indolent enlargement, a pattern that was inconsistent with the expected behavior of metastatic disease. Because of this clinicoradiologic discrepancy, thoracoscopic wedge resection was performed for definitive diagnosis. Histopathology revealed acinar-predominant invasive adenocarcinoma of the lung that was positive for TTF-1, and Napsin A, and negative for ALK. Next-generation sequencing identified an EGFR exon 20 insertion (p.N771_P772insG) and a low tumor mutational burden. The discordant clinical behavior, isolated and indolent radiologic evolution, divergent histopathologic and immunophenotypic profiles, and distinct oncogenic drivers collectively supported a diagnosis of synchronous primary tumors rather than metastatic IMT. Because sequencing was limited to the DICER1 hotspot region, other hereditary cancer-predisposition mechanisms could not be definitively excluded. This case underscores the importance of longitudinal imaging, integrated pathology, and molecular profiling when presumed metastatic disease follows an atypical course pediatric oncology.

## 1 Introduction

Inflammatory myofibroblastic tumor (IMT) is a mesenchymal neoplasm of intermediate malignant potential, characterized by proliferation of myofibroblastic spindle cells with an inflammatory infiltrate (1–3). The 2020 WHO classification recognizes IMT as an intermediate tumor that may rarely metastasize. Proposed etiologic factors include trauma, autoimmunity, and viral infection (4–6). Genetically, more than half of IMTs harbor ALK gene rearrangements (7), whereas ROS1 and NTRK fusions have been identified in a subset of ALK-negative cases (8–10). Definitive diagnosis of laryngeal IMT requires histopathologic confirmation, ideally accompanied by molecular testing to identify actionable kinase fusions that may guide targeted therapy (11). IMT predominantly affects children and adolescents and most often arises in thoracic and abdominopelvic sites, although head and neck involvement has also been reported (12–14). Pediatric laryngeal IMT in children was first described by Munoz et al. in 2001 (15); to date, 11 pediatric cases have been reported, with recurrence documented in 4 of 10 evaluable patients experiencing recurrence (Supplementary Table 1).

Primary lung adenocarcinoma is classified into papillary, acinar, and tubular subtypes (16) but is exceedingly rare in children, with only 62 pediatric cases documented in a 2018 systematic review (17, 18). Its etiology remains unclear and may involve genetic alterations (19) or congenital lung malformations (20), while tobacco exposure is considered largely irrelevant in pediatric patients (21). Diagnosis is challenging because radiologic appearances are variable, and confirmation requires histopathologic assessment.

The simultaneous development of two histogenetically and genetically distinct primary tumors to develop simultaneously, such as mesenchymal IMT and epithelial lung adenocarcinoma, is exceptionally rare. To the best of our knowledge, synchronous ALK-positive IMT and EGFR-mutant lung adenocarcinoma have not previously been reported in a child. We describe a case in which the clinical behavior prompted reassessment of the initial diagnosis, ultimately leading to the identification of synchronous primary tumors through integrated pathologic and genomic evaluation.

## 2 Case description

A 7-year-old boy was admitted with a history of hoarseness and dyspnea for more than six months. Six months before the present admission, he initially developed exertional dyspnea, and fiberoptic laryngoscopy suggested a possible subglottic papilloma. Contrast-enhanced CT showed an abnormal dense shadow in the anterior subglottic tracheal space, and endoscopic resection of the presumed laryngeal papilloma was performed. Histopathologic examination demonstrated IMT. Postoperative PET-CT subsequently revealed a suspected metastatic focus in the right lower lobe. He then received VDC, IE, and VIP chemotherapy regimens for five cycles in total, achieving stable disease.

**Figure 1:**
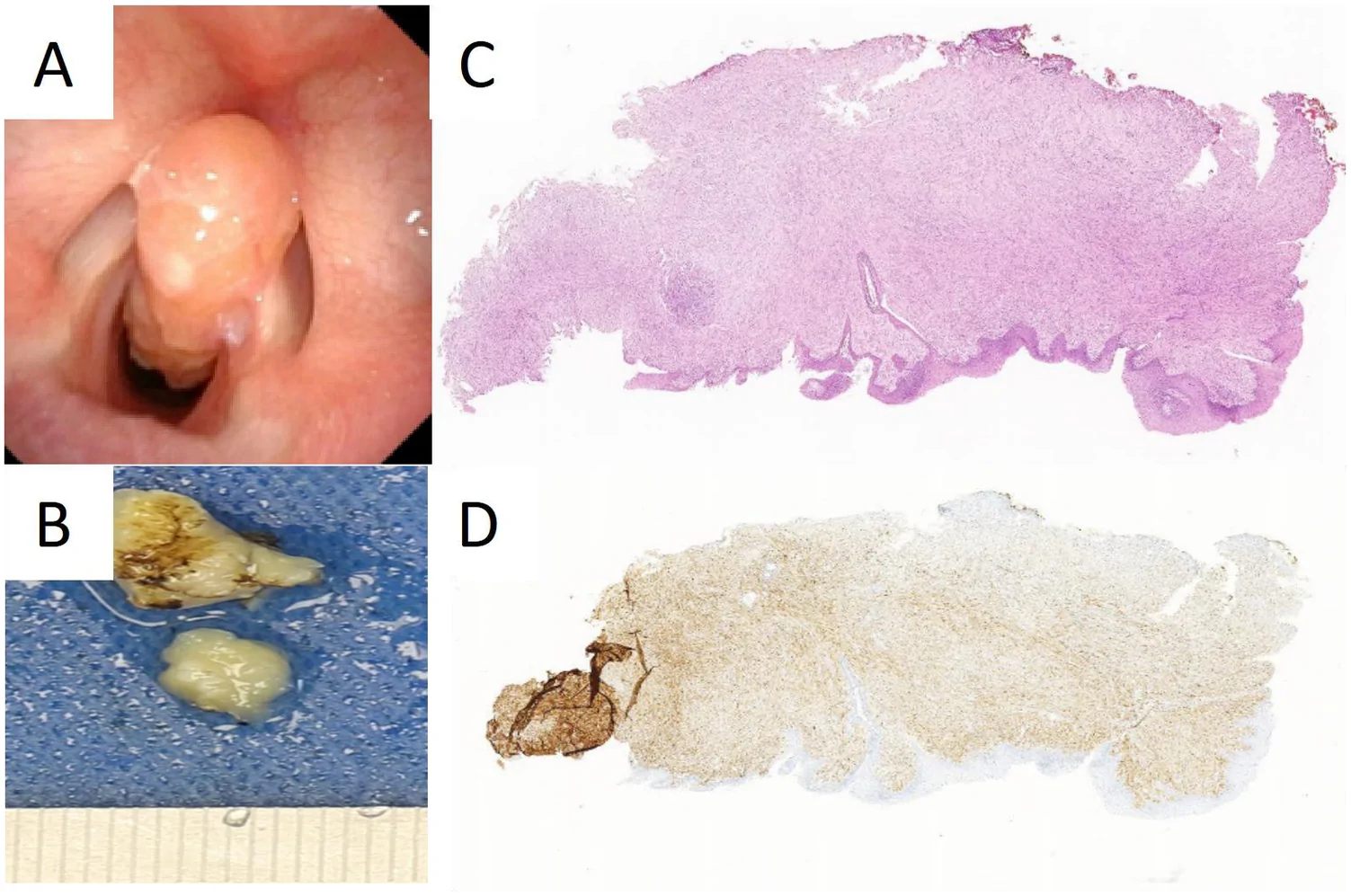
Recurrent laryngeal lesion. **(A)** Laryngoscopy showing recurrence six months after the initial diagnosis, with adenoid hypertrophy in the nasopharynx. **(B)** Gross appearance of the resected laryngeal lesion. **(C)** Hematoxylin and eosin staining showing spindle cell proliferation with inflammatory infiltration (original magnification, ×200). **(D)** ALK immunohistochemical staining showing strong cytoplasmic positivity in spindle cells (original magnification, ×200).

At the present admission, a complete blood count showed decreased red blood cell count and hemoglobin level, as well as a reduced absolute CD3 + CD4+ T-lymphocyte count. Alpha-fetoprotein, carcinoembryonic antigen (1.02 ng/mL; normal range, 0–5 ng/mL), and neuron-specific enolase levels were within normal limits. Laryngoscopy revealed a laryngeal mass with adenoid hyperplasia (Figure 1A), and laryngoscopy-guided mass resection was performed. Postoperative histopathology was consistent with recurrent or residual inflammatory myofibroblastic tumor. Immunohistochemistry showed positivity for ALK (OTI1H7) and ALK (5A4) (Figures 1B–D).

**Figure 2:**
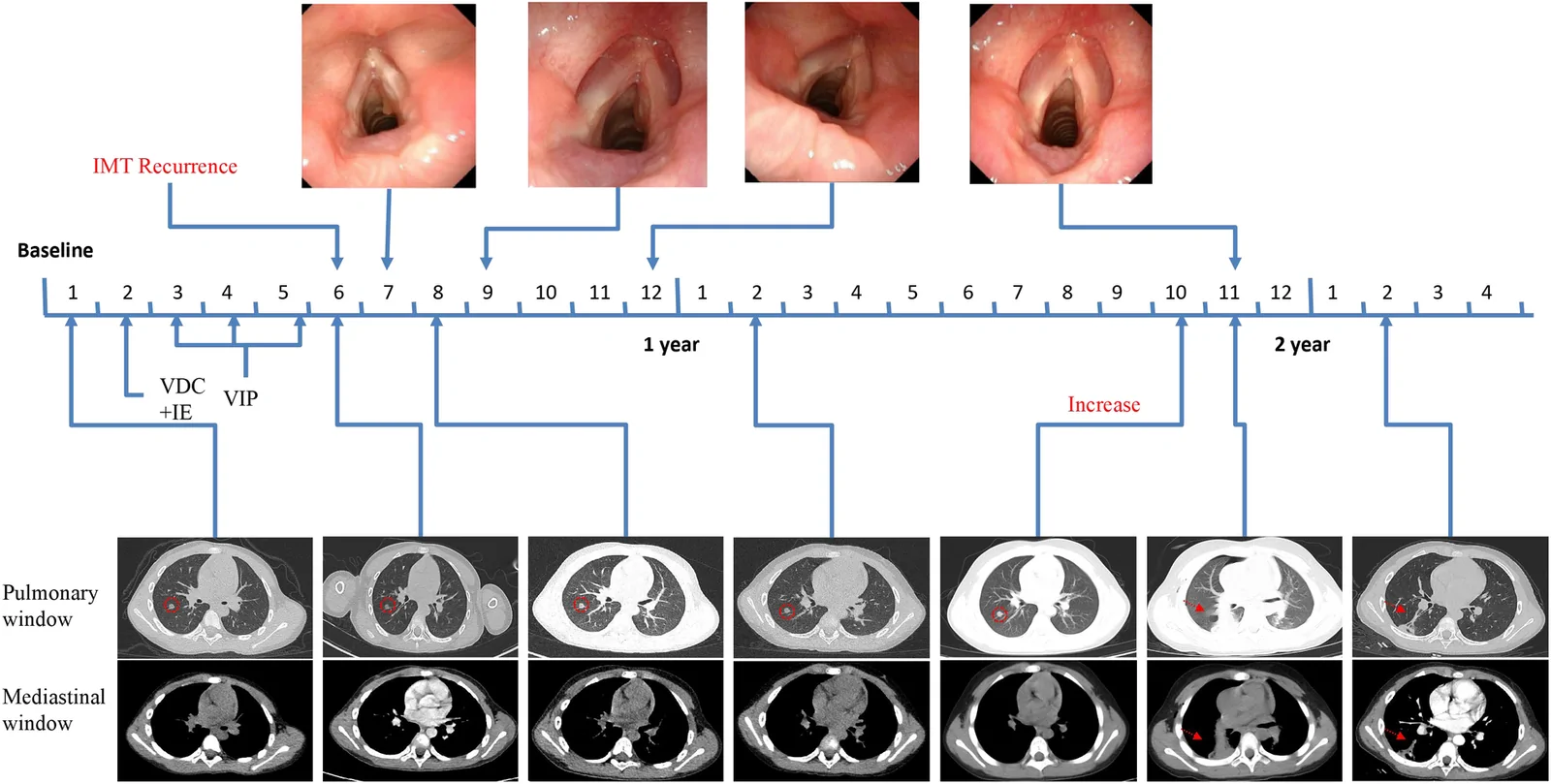
Treatment timeline and longitudinal radiologic evolution of the lesion. (The initial diagnosis and PET-CT scan were performed at other institutions; the relevant records are unavailable. This figure only records events documented at our hospital).

**Figure 3:**
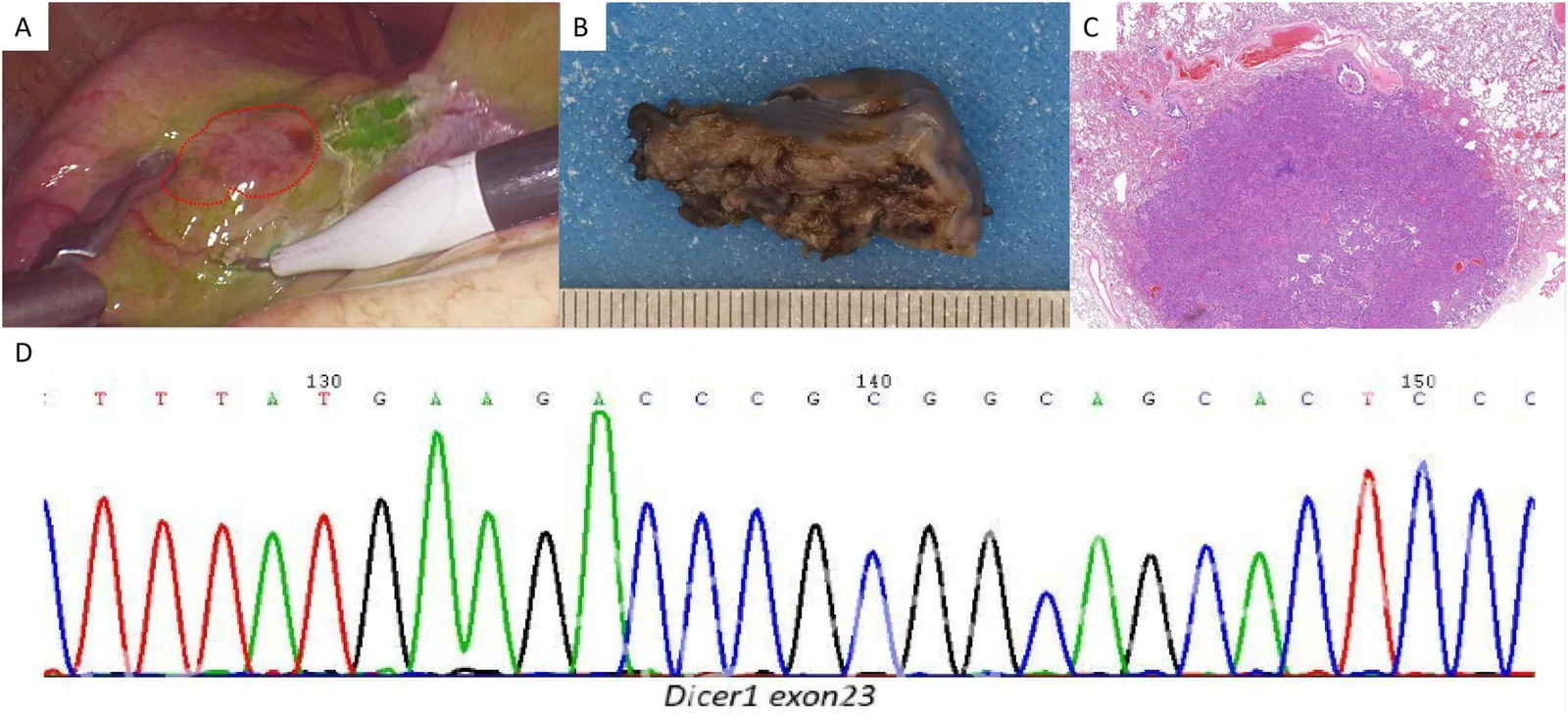
Pathologic findings of the pulmonary lesion. **(A)** Intraoperative thoracoscopic image of the lesion. **(B)** Gross appearance of the resected specimen. **(C)** Hematoxylin and eosin staining showing invasive adenocarcinoma with acinar-predominant pattern (original magnification, ×200). **(D)** Targeted sequencing of the DICER1 hotspot region (exons 22–24) showing no pathogenic variants.

Routine follow-up laryngoscopy showed no evidence of recurrence (Figure 2); however, chest CT demonstrated progressive enlargement of the pulmonary nodule (Figure 2). At 22 months after the nodule was first detected, the chest CT showed a well-circumscribed solid nodule measuring approximately 0.8 × 0.8 cm in the dorsal segment of the right lower lobe with clear boundaries. The patient was readmitted, and then underwent fluorescence-guided thoracoscopic wedge resection of the right lower lobe lesion (Figures 3A,B). The resected specimen was initially described as a predominantly epithelial, papillary tumor. Final pathologic examination of the resected specimen confirmed acinar-predominant invasive lung adenocarcinoma, acinar-predominant type. According to the 8th edition of the AJCC staging system, the pathologic stage was pT1aN0Mx because nodal sampling was not performed during wedge resection. The resection margins were negative. Lymphovascular invasion and spread through air spaces (STAS) were not identified, and mitotic activity was low (<1 per 10 high-power fields). Immunohistochemistry showed positivity for pan-cytokeratin, EMA, CK7, TTF-1 (8G7G3/1), and Napsin A, with diffuse p53 expression (Figure 3C). Because the lesion was early-stage and completely resected, no adjuvant therapy was administered. Subsequent next-generation sequencing identified an EGFR exon 20 insertion (p.N771_P772insG), with a low tumor mutational burden (0.55 mutations/Mb). CT performed immediately after surgery and at three months postoperatively showed no evidence of recurrence. The patient remains under long-term follow-up.

## 3 Discussion

The management of this case was shaped by two interrelated challenges: achieving local control of recurrent laryngeal IMT in an anatomically constrained site and resolving the diagnostic uncertainty surrounding an indolent pulmonary nodule. The final diagnosis of synchronous primary tumors required systematic re-evaluation of an initially plausible metastatic hypothesis. These issues are discussed below.

The clinical manifestations of laryngeal IMT are similar to those at other anatomic sites and primarily reflect local mass effect (22), most commonly hoarseness and dyspnea. Systemic manifestations, including weight loss, pain, fever, and elevated blood cell counts, may also occur (3). Complete surgical resection remains the preferred treatment for laryngeal IMT. In our review of the 11 previously reported pediatric laryngeal IMT cases (Supplementary Table 1), 4 of 10 evaluable patients (40%) experienced at least one recurrence, underscoring the difficulty of achieving durable local control despite apparently complete resection. This finding is broadly consistent with the 25% recurrence rate reported in larger IMT cohorts, while highlighting the anatomic challenges specific to the larynx.

Previous studies have reported a 25% recurrence rate for IMT (23), primarily attributed to incomplete resection and tumor size. However, a recent study by Rich et al. found no significant association between margin status (negative, microscopically positive, or macroscopically positive) and event-free survival (24). For unresectable or advanced disease, tyrosine kinase inhibitors (TKIs) targeting oncogenic drivers such as ALK, including crizotinib, have become central to systemic treatment (11). As the first approved ALK-TKI, crizotinib has shown high response rates in ALK-positive IMT. Tang et al. summarized the use of different generations of ALK-TKIs in ALK-positive inflammatory myofibroblastic tumors, including cases harboring rare or complex ALK fusions (25). Although our patient initially received chemotherapy and achieved disease stabilization, current evidence supports ALK-TKIs, particularly crizotinib, as the preferred systemic option for patients with advanced or unresectable ALK-positive IMT. The current status of ALK inhibitor use in pediatric patients is summarized in Supplementary Table 2.

Regarding the pulmonary nodule, the initial working diagnosis of metastatic disease from laryngeal IMT was clinically reasonable. First, the patient had an established diagnosis of IMT, a tumor with recognized metastatic potential, albeit at a low rate (<5%). Second, in keeping with diagnostic parsimony, and given the extreme rarity of a second primary lung adenocarcinoma in a 7-year-old child, the likelihood of a synchronous primary malignancy was initially considered low. Third, the nodule was located in the peripheral lower lobe, a distribution compatible with pulmonary involvement by IMT. Although the lesion was visible on lung-window images but not on mediastinal-window images, this finding was nonspecific; therefore, metastatic IMT was initially favored. Possible differential diagnoses: inflammatory nodule (to be ruled out), early-stage lung adenocarcinoma (to be considered).

However, the metastatic hypothesis was ultimately refuted by four converging lines of evidence. First, the clinical course was atypical: the nodule showed very slow growth over approximately 22 months, which was inconsistent with the expected behavior of metastatic IMT. Second, the imaging evolution argued against metastasis: serial high-resolution CT scans over two years demonstrated a single, well-defined, isolated nodule with gradual, almost linear enlargement and no evidence of multifocal dissemination; its persistent absence on mediastinal-window images further prompted reconsideration of the initial interpretation. Third, the histopathologic and immunophenotypic profiles indicated distinct cellular origins: the laryngeal lesion showed mesenchymal spindle-cell features with strong ALK positivity, whereas the pulmonary lesion was an epithelial adenocarcinoma that was positive for TTF-1 and Napsin A and negative for ALK. Finally, genomic profiling demonstrated separate oncogenic drivers: the laryngeal IMT harbored a THBS1-ALK fusion, whereas the lung adenocarcinoma harbored an EGFR exon 20 insertion (p.N771_P772insG) without an ALK rearrangement. Taken together, these findings excluded metastatic IMT and supported the diagnosis of synchronous primary tumors.

There is no consensus on the optimal management of pediatric invasive lung cancer. Treatment is generally extrapolated from adult practice and individualized according to tumor stage, histology, and molecular findings (26). Surgery remains the mainstay of treatment for early-stage lung adenocarcinoma, and wedge resection may be appropriate for small peripheral lesions when complete excision can be achieved. In adults with advanced lung adenocarcinoma, targeted therapies directed against actionable drivers such as EGFR or ALK have substantially improved outcomes. In contrast, evidence-based targeted treatment strategies for pediatric lung adenocarcinoma remain limited.

The identification of an EGFR exon 20 insertion in this case is particularly noteworthy. Unlike classic sensitizing EGFR variants, such as exon 19 deletions and L858R, exon 20 insertions account for approximately 4%–12% of EGFR-mutant NSCLC cases (27, 28) and are associated with primary resistance to first-, second-, and third-generation EGFR TKIs. This resistance is largely attributable to structural alterations that stabilize the *α*C-helix in an active conformation and create steric hindrance within the drug-binding pocket (29, 30). Nevertheless, the therapeutic landscape for this molecular subgroup has evolved rapidly in recent years. Amivantamab, a bispecific EGFR-MET antibody, has emerged as a key treatment option, and the phase III PAPILLON trial established amivantamab plus platinum-based chemotherapy as a first-line standard of care (median progression-free survival, 11.4 vs. 6.7 months; hazard ratio, 0.40). A subcutaneous formulation was approved in December 2025, offering comparable efficacy with markedly fewer infusion-related reactions (27). Sunvozertinib, a highly selective oral EGFR TKI, received FDA accelerated approval in July 2025 for platinum-pretreated patients on the basis of the WU-KONG1 trial (objective response rate, 54.3%; median duration of response, 11.1 months) (28, 31). Other promising agents in late-stage development include zipalertinib (REZILIENT1: objective response rate, 38.4%; retained activity after amivantamab, 39%) and furmonertinib (FAVOUR: objective response rate up to 69% in treatment-naive patients, with notable CNS penetration). In contrast, mobocertinib, the first FDA-approved oral TKI for EGFR exon 20 insertion-mutant NSCLC, was voluntarily withdrawn in October 2023 after the negative phase III EXCLAIM-2 trial (27). Collectively, these developments underscore the rapidly changing treatment landscape for EGFR exon 20 insertion-mutant NSCLC and highlight the importance of comprehensive molecular profiling to guide precision therapy. However, to the best of our knowledge, no dedicated pediatric trial has evaluated EGFR exon 20 insertion-directed therapy in children with lung adenocarcinoma. In pediatric bithalamic glioma, a distinct brain tumor entity, EGFR exon 20 insertions occur in up to 85% of cases, and treatment with EGFR or MEK inhibitors prolonged median overall survival from 13.0 to 21.5 months (*p* = 0.010) (32). Although this evidence arises from a different histologic context, it suggests that EGFR exon 20 insertions may be therapeutically targetable in pediatric patients.

The patient presented with two primary malignancies, namely IMT, a low-grade mesenchymal tumor, and invasive lung adenocarcinoma, a malignant epithelial tumor, diagnosed within six months of each other. The postoperative diagnosis was therefore revised to synchronous dual primary tumors (33). The occurrence of two rare primary tumors in a 7-year-old child naturally raises concern for an underlying hereditary cancer-predisposition syndrome. Beyond DICER1, several germline alterations are associated with multiple tumor types in children, including TP53 (Li-Fraumeni syndrome), PTEN (Cowden syndrome), NF1 (neurofibromatosis type 1), and RB1 (retinoblastoma). To explore this possibility, we performed targeted sequencing of the DICER1 hotspot region (exons 22–24), given its established association with rare pediatric lung tumors such as pleuropulmonary blastoma. No pathogenic variants were identified (Figure 3D). However, this targeted approach does not exclude other germline predisposition mechanisms. Comprehensive germline testing or whole-exome sequencing was not performed because of the retrospective nature of this case report. Therefore, although the absence of DICER1 mutations and the mutually exclusive somatic driver alterations in the two tumors argue against a shared clonal origin, we cannot definitively conclude that this case is sporadic. The possibility of an unrecognized germline predisposition cannot be entirely excluded, and lifelong surveillance and genetic counseling are recommended for the patient and his family. The incidence of synchronous primary malignancies in children is unknown but presumed to be exceedingly low. By contrast, the prevalence of germline pathogenic variants in pediatric cancer-predisposition syndromes is estimated at 8%–10% across unselected childhood cancers, although rates vary according to tumor type and selection criteria (34).

## 4 Patient perspective

The patient's parents expressed satisfaction with the overall diagnostic and treatment process. Although the period of diagnostic uncertainty was difficult for their child and family, they were reassured that the final diagnosis was two primary tumors rather than metastatic disease. They also expressed trust in the clinical team's decision-making and recognized the importance of genomic testing in guiding individualized management. At the most recent follow-up, the patient had returned to school and normal daily activities.

## 5 Conclusion

To the best of our knowledge, this is the first reported case of synchronous ALK-positive laryngeal IMT and EGFR-mutant lung adenocarcinoma in a pediatric patient. The case illustrates that when the clinical trajectory diverges from expectation, as exemplified by the indolent growth of the pulmonary nodule over 22 months, the assumption of metastasis should be rigorously re-examined. It also demonstrates that an integrated diagnostic approach combining longitudinal imaging, detailed histopathologic assessment, immunophenotyping, and NGS profiling is essential for establishing the correct diagnosis and informing future therapeutic decisions.

## Data Availability

The datasets presented in this study can be found in online repositories. The names of the repository/repositories and accession number(s) can be found in the article/Supplementary Material.
