# Supplementary material for: Case Report: Synchronous ALK-positive laryngeal inflammatory myofibroblastic tumor and EGFR-mutant lung adenocarcinoma in a child
Source: Front Pediatr. 2026 Aug 6;14:1874110. doi: 10.3389/fped.2026.1874110 (PMC13490079; doi:10.3389/fped.2026.1874110)
Supplement: Supplementary file 1 [file Table1.pdf]

Table 1.Series of Pediatric Laryngeal IMT Cases.

Number	Authors	Age(y)/sex	Performance	Treatment	ALK status	Recurrence	References
1	Tay et al	12/F	Hoarseness (lasting 9 months)	Endoscopic complete resection	N	N	[1]
2	Hussein et al	13/M	Intermittent hoarseness, shortness of breath after exertion	Tracheotomy under local anesthesia + CO_2 laser resection under direct laryngoscopy	N	N	[2]
3	He et al	10/M	Hoarse for a year	Between 2010 and 2011, the patient underwent five surgeries. The first three were misdiagnosed, with the fourth surgery yielding a pathological diagnosis of IMT. The fifth surgery employed intraoperative frozen section analysis to ensure negative surgical margins.	+	Y	[3]
4	Michael et al	2/M	Snoring, sleep apnea	Initial surgery: Removal of the main tumor mass only Secondary surgery: Laser partial supraglottic laryngectomy	N	Y	[4]
5	Liu et al	7/M	Inspiratory dyspnea	Tracheostomy + endoscopic tumor resection. Recurrence at 2 months postoperatively; treated with endoscopic holmium laser thermal ablation.	N	Y	[5]
6	Alkheder et al	10/F	Progressive dyspnea + exertional wheezing, lasting 18 months	Complete resection under endoscopy.	+(Focal)	N	[6]
7	Monica et al	5/F	Persistent cough, shortness of breath, wheezing for 4 months	Complete excision using KTP laser (3W, continuous mode, total energy 984J)	N	N	[7]
8	Munoz et al	5/M	Shortness of breath, episodes of inspiratory wheezing for 3 months	Complete resection + autologous perichondrial grafting	N	-	[8]
9	Hanna et al	7/M	Progressive wheezing for 2 weeks	Endoscopic diode laser ablation without tracheal intubation. Postoperative intravenous and oral steroids administered. Follow-up treatment included three laryngoscopic laser procedures within 8 months.	N	N	[9]
10	Sudesh et al	10/M	Hoarse voice for 6 months	Complete resection under general anesthesia via microscopic laryngoscopy	N	N	[10]
11	Zhang et al	8/M	Coughing, wheezing, shortness of breath, worsening with activity	The patient underwent bronchoscopic tumor ligation and resection, but residual tumor persisted and invaded the entire thickness of the trachea. Subsequently, a tracheal resection and reconstruction procedure was performed under spontaneous breathing without intubation.	N	Y	[11]

Table 2. ALK Inhibitors and EGFR Targeted Therapies Commonly Used in Children.

Name	Category	Indications	Dosage	Therapeutic Efficacy	Adverse Drug Reactions
Crizotinib[12]	First-generation ALK inhibitors	Recurrent/refractory, unresectable ALK-positive ALCL and IMT in patients younger than 22 years of age	100 mg/m² (twice daily, 1 patient)<br>165 mg/m² (twice daily, 1 patient)<br>280 mg/m² (twice daily, 12 patients)	Overall Response Rate (ORR): 86% (12/14 patients)<br>Complete Response Rate (CR): 36% (5/14 patients)<br>Partial Response Rate (PR): 50% (7/14 patients)<br>Median Duration of Treatment: 1.63 years	71% of patients experienced at least one Grade ≥3 adverse event.<br>57% of patients experienced Grade ≥3 adverse events related to the study drug.<br>Most common adverse reactions: Decreased neutrophil count
Ceritinib[13]	Second-generation ALK inhibitors	ALK-positive malignant tumors (including IMT) in patients aged 1–18 years with advanced/metastatic disease who have failed standard therapy	Recommended dosage: 500 mg/m² once daily, taken with food	Overall Response Rate (ORR): 70% (7/10 patients)<br>Complete Response Rate (CR): 0%<br>Partial Response Rate (PR): 70% (7/10 patients)<br>Disease Control Rate (DCR): 80% (including PR and disease stabilization)<br>Median follow-up time: 33.2 months	81% of patients experienced at least one ≥ Grade 3 adverse event.<br>The incidence of serious adverse events was 48%, with 37% being Grade 3-4.<br>The most common adverse reactions (all grades): vomiting (87%), diarrhea (78%), etc.<br>The most common Grade 3-4 treatment-related adverse reactions: elevated alanine aminotransferase (39%).
Lorlatinib[14]	Third-generation ALK/ROS1 macrolide inhibitor	ALK-Driven Relapsed/Refractory Neuroblastoma	Single-agent and combination chemotherapy in pediatric patients<br>RP2D: 115 mg/m²/day once daily	Pediatric monotherapy (<18 years): CR/PR rate 13%; modified remission rate 30% (CR/PR/MR)<br>Pediatric combination chemotherapy (<18 years): CR/PR rate 25%; modified remission rate 63%	Most common: Hypertriglyceridemia (90%), weight gain (87%), hypercholesterolemia (79%)
Entrectinib[15]	TRK/ROS1/ALK Multi-Target Inhibitor	Pediatric patients aged 1 month and older with NTRK fusion-positive, recurrent, or refractory solid tumors	---	Overall Response Rate (ORR): 57% (95% CI: 43–71).<br>Complete Response Rate (CR): 7%.<br>Duration of response: 2.8 months to 26.0+ months.<br>Durability: 68% of responses lasted ≥ 6 months.	Most severe toxicity: congestive heart failure, central nervous system effects, etc.
Nimotuzumab[16]	Humanized anti-epidermal growth factor receptor monoclonal antibody, an EGFR inhibitor.	Adjuvant or additional therapy for children and adolescents diagnosed with glioma at age ≤ 21	---	Overall survival: Median OS range 3.2 to 22.8 months.<br>Progression-free survival: Median PFS range 1.7 to 21.6 months.	The incidence of serious adverse events was low, including: hematologic toxicity, fever, acute respiratory infections, etc.

[1] S.Y. Tay, and A. Balakrishnan, Laryngeal inflammatory myofibroblastic tumor (IMT): a case report and review of the literature. J Med Case Rep 10 (2016) 180.

[2] H. Smaily, P. Cherfane, and N. Matar, Pediatric laryngeal inflammatory myofibroblastic tumour: Case report and systematic review of the literature. Auris Nasus Larynx 48 (2021) 1047-1053.
[3] C.Y. He, G.H. Dong, and H.G. Liu, Recurrent laryngeal inflammatory myofibroblastic tumor with positive anaplastic lymphoma kinase mimicking recurrent respiratory papillomatosis: a case report. World J Surg Oncol 12 (2014) 54.
[4] M. Rodrigues, R.J. Taylor, C.C. Sun, and J.S. Wolf, Inflammatory myofibroblastic tumor of the larynx in a 2-year-old male. ORL J Otorhinolaryngol Relat Spec 67 (2005) 101-5.
[5] F. Liu, Y. Qin, Z. Zhang, M. Li, B. Feng, W. Ding, and S. Dong, Therapeutic strategy and prognostic analysis of inflammatory myofibroblastic tumor in the head and neck: a retrospective study. PeerJ 13 (2025) e19315.
[6] A. Alkheder, Y. Al-Ghabra, A. Azar, A. Dmirieh, A.B.A. Mohsen, and A. Yousfan, Subglottic Inflammatory Myofibroblastic Tumor in a Child: A Rare Case Report. Ear Nose Throat J (2025) 1455613251353637.
[7] M.Q. Kieu, P.J. Thottam, V. DaCosta, L. Gonzalez-Krellwitz, J.M. Poulik, and D.N. Madgy, Treatment of inflammatory myofibroblastic tumor of the subglottis with KTP laser: a case report. J Voice 28 (2014) 841 e1-4.
[8] A. Munoz, and M. Villafruela, Inflammatory pseudotumor of the larynx: MR findings in a child. Pediatr Radiol 31 (2001) 459-60.
[9] S.J. Hanna, E. Blenke, R. Sharma, and L.C. Knight, Laryngeal inflammatory pseudotumour: an unusual cause of airway obstruction. Int J Pediatr Otorhinolaryngol 69 (2005) 1253-5.
[10] S. Kumar, A.K. Gupta, and N. Kakkar, Inflammatory myofibroblastic tumor larynx mimicking laryngeal papillomatosis. International Journal of Pediatric Otorhinolaryngology Extra 4 (2009) 42-44.
[11] Y. Zhang, Z. Mo, C. Yang, J. He, S. Li, and L. Lan, Non-intubated tracheal resection and reconstruction for a tracheal tumor in an 8-year-old child. J Cardiothorac Surg 19 (2024) 468.
[12] Y.P. Mosse, S.D. Voss, M.S. Lim, D. Rolland, C.G. Minard, E. Fox, P. Adamson, K. Wilner, S.M. Blaney, and B.J. Weigel, Targeting ALK With Crizotinib in Pediatric Anaplastic Large Cell Lymphoma and Inflammatory Myofibroblastic Tumor: A Children's Oncology Group Study. J Clin Oncol 35 (2017) 3215-3221.
[13] M. Fischer, L. Moreno, D.S. Ziegler, L.V. Marshall, C.M. Zwaan, M.S. Irwin, M. Casanova, C. Sabado, B. Wulff, M. Stegert, L. Wang, F.K. Hurtado, F. Branle, B. Geoerger, and J.H. Schulte, Ceritinib in paediatric patients with anaplastic lymphoma kinase-positive malignancies: an open-label, multicentre, phase 1, dose-escalation and dose-expansion study. Lancet Oncol 22 (2021) 1764-1776.
[14] K.C. Goldsmith, J.R. Park, K. Kayser, J. Malvar, Y.Y. Chi, S.G. Groshen, J.G. Villablanca, K. Krytska, L.M. Lai, P.T. Acharya, F. Goodarzian, B. Pawel, H. Shimada, S. Ghazarian, L. States, L. Marshall, L. Chesler, M. Granger, A.V. Desai, R. Mody, D.A. Morgenstern, S. Shusterman, M.E. Macy, N. Pinto, G. Schleiermacher, K. Vo, H.C. Thurm, J. Chen, M. Liyanage, G. Peltz, K.K. Matthay, E.R. Berko, J.M. Maris, A. Marachelian, and Y.P. Mosse, Lorlatinib with or without chemotherapy in ALK-driven refractory/relapsed neuroblastoma: phase 1 trial results. Nat Med 29 (2023) 1092-1102.
[15] L. Marcus, M. Donoghue, S. Aungst, C.E. Myers, W.S. Helms, G. Shen, H. Zhao, O. Stephens, P. Keegan, and R. Pazdur, FDA Approval Summary: Entrectinib for the Treatment of NTRK gene Fusion Solid Tumors. Clin Cancer Res 27 (2021) 928-932.

[16] M.A. Parenrengi, W. Suryaningtyas, A. Al Fauzi, A. Hafid Bajamal, K. Kusumastuti, B. Utomo, A. Muslim Hidayat Thamrin, and B. Sulistiono, Nimotuzumab as Additional Therapy for GLIOMA in Pediatric and Adolescent: A Systematic Review. Cancer Control 29 (2022) 10732748211053927.
